# Comprehensive genetic assessment of a functional TLR9 promoter polymorphism: no replicable association with asthma or asthma-related phenotypes

**DOI:** 10.1186/1471-2350-12-26

**Published:** 2011-02-15

**Authors:** Nancy E Lange, Xiaobo Zhou, Jessica Lasky-Su, Blanca E Himes, Ross Lazarus, Manuel Soto-Quirós, Lydiana Avila, Juan C Celedón, Catherine M Hawrylowicz, Benjamin A Raby, Augusto A Litonjua

**Affiliations:** 1 Channing Laboratory, Brigham and Women's Hospital and Harvard Medical School, 181 Longwood Avenue, Boston, MA, 02115, USA; 2 Division of Pulmonary and Critical Care Medicine, Brigham and Women's Hospital and Harvard Medical School, 75 Francis Street, Boston, MA, 02115, USA; 3 Division of Pediatric Pulmonology, Hospital Nacionál de Niños, Calle 20, San José, Costa Rica; 4 Division of Pediatric Pulmonary Medicine, Allergy and Immunology, Children's Hospital of Pittsburgh of the University of Pittsburgh Medical Center, One Children's Hospital Drive, 4401 Penn Ave., Pittsburgh, PA, 15224, USA; 5 Department of Asthma, Allergy and Respiratory Science, King's College London, Guy's Hospital, Great Maze Pond, London SE1 9RT, UK

## Abstract

**Background:**

Prior studies suggest a role for a variant (rs5743836) in the promoter of toll-like receptor 9 (TLR9) in asthma and other inflammatory diseases. We performed detailed genetic association studies of the functional variant rs5743836 with asthma susceptibility and asthma-related phenotypes in three independent cohorts.

**Methods:**

rs5743836 was genotyped in two family-based cohorts of children with asthma and a case-control study of adult asthmatics. Association analyses were performed using chi square, family-based and population-based testing. A luciferase assay was performed to investigate whether rs5743836 genotype influences TLR9 promoter activity.

**Results:**

Contrary to prior reports, rs5743836 was not associated with asthma in any of the three cohorts. Marginally significant associations were found with FEV_1 _and FVC (p = 0.003 and p = 0.008, respectively) in one of the family-based cohorts, but these associations were not significant after correcting for multiple comparisons. Higher promoter activity of the CC genotype was demonstrated by luciferase assay, confirming the functional importance of this variant.

**Conclusion:**

Although rs5743836 confers regulatory effects on TLR9 transcription, this variant does not appear to be an important asthma-susceptibility locus.

## Background

Asthma is a public health problem of considerable importance with over 300 million people affected worldwide[1]. Asthma is likely due to genetic and environmental determinants that are poorly understood. Although primarily a disorder resulting from TH2 cell mediated inflammation, significant interest is developing in investigating innate immunity in the search to understand the pathogenesis of this disease[2].

Toll-like receptors (TLRs) are evolutionarily conserved components of the innate immune system that act to directly recognize pathogen-derived elements, including viral and bacterial DNA, lipopolysaccharides, and proteoglycans. They provide a crucial link between the recognition of pathogens by the innate immune system and subsequent activation of adaptive immunity, inducing maturation of antigen presenting cells and differentiation of T cells. TLR9 is expressed in plasmacytoid dendritic cells, B cells, CD3+CD4+ T cells and pulmonary epithelial cells[3], and recognizes bacterial unmethylated cytosine-guanine dinucleotide (CpG) motifs and viral antigens[4]. When activated it leads to maturation of dendritic cells and release of pro-inflammatory TH1 cytokines such as IL-6, IL-12, TNF, IFN-γ and IFN-α. It can also be counter-regulatory to the induction of T regulatory cells (Tregs) and release of the anti-inflammatory cytokine IL-10.

Our group and others have previously reported an association between a single nucleotide polymorphism (SNP) in the promoter region of TLR9 (rs5743836 or -1237C/T) and asthma in European American adults[5] and Tunisian children[6]. Other studies have shown associations with other inflammatory diseases such as allergic bronchopulmonary aspergillosis (ABPA)[7], atopic eczema[8], and inflammatory bowel disease (IBD)[9-11]. The observed associations between rs5743836 and various immune-mediated diseases in different populations point to the possibility that this SNP may be functional and potentially involved in immune system regulation. Of interest, an interaction between TLR9 and vitamin D has been implicated in the induction of Tregs that produce IL-10[12], an anti-inflammatory cytokine with potent inhibitory effects on TH1 and TH2 responses[13].

Given previous findings, we sought to investigate the possible association between SNP rs5743836 and asthma in three different cohorts of asthmatics: the Childhood Asthma Management Program (CAMP), the Genetics of Asthma in Costa Rica study (Costa Rica), and the Informatics for Integrating Biology to the Bedside (i2b2) Crimson Asthma Project (iCAP). CAMP is a well-characterized cohort of children from North America with mild to moderate asthma in whom we have multiple measures of lung function as well as data on exacerbations, atopy and vitamin D levels. Because of the extensive phenotyping in CAMP, we decided to probe more deeply in this cohort by investigating possible associations with asthma-related quantitative phenotypes, and to assess for interaction with vitamin D. In addition, we performed functional studies in cell cultures to determine potential functional significance of this SNP.

## Methods

### Population and phenotyping data

#### CAMP cohort

The Childhood Asthma Management program (CAMP) is a multi-center, randomized, double-masked, placebo-controlled clinical trial designed to investigate the long-term effects of inhaled anti-inflammatory medications in children with mild to moderate asthma[14,15]. Of the 1041 children enrolled in the original clinical trial, DNA samples were obtained from 968 participating children and 1518 of their parents. Due to the small sample size of other ethnic groups, this analysis was restricted to 711 self-reported non-Hispanic white probands (n = 711) and available parents (n = 638 complete trios). A diagnosis of asthma was based on methacholine hyperreactivity (PC_20 _*≤ *12.5 gm/ml)and one or more the following criteria for at least 6 months in the year before recruitment: 1) asthma symptoms at least 2 times per week, 2) at least 2 uses per week of an inhaled bronchodilator, and 3) daily asthma medication. Spirometry was performed according to American Thoracic Society (ATS) recommendations [16] using a volume displacement spirometer. Vitamin D levels (25-hydroxyvitamin D) from serum collected prior to treatment randomization and at 44 months post-randomization were measured using a radionimmunoassay method in Dr. Bruce Hollis' laboratory at the Medical University of South Carolina [17,18]. The Institutional Review Board of each of the CAMP study centers approved this study. Informed assent and consent were obtained from all study participants and their parents to collect DNA for genetic studies.

#### Costa Rica cohort

Participants in the Costa Rica study included 616 children ages 6-14 years with asthma (defined as physician-diagnosed asthma and ≥ 2 respiratory symptoms or recurrent asthma attacks in the past year) recruited between February 2001 and March 2005, as previously described[19]. This population is a genetic isolate of mixed Spanish and Amerindian descent with one of the world's highest rates of asthma (27.4% of children aged 6-7 years)[20]. The study was approved by the Institutional Review Boards of the Hospital Nacionál de Niños (San Jose, Costa Rica) and Brigham and Women's Hospital (Boston, MA, USA). After obtaining written informed consent from the parents of participating children, each participant completed a modified version of the questionnaire used in the Collaborative Study on the Genetics of Asthma[21], translated into Spanish. Each subject performed baseline spirometry according to ATS recommendations[16], using a Collins Survey Tach Spirometer (Warren Collins, Braintree, MA, USA). Following baseline spirometry, subjects received 200 μg of albuterol. Spirometry was repeated after 15 minutes.

#### iCAP cohort

The i2b2 Crimson Asthma Project (iCAP) consists of Partners Healthcare System, Inc. (Boston, MA) patients who were selected based on extracted de-identified electronic medical record (EMR) data and whose DNA was obtained via discarded clinical samples. The collection and study of this data was approved by the Institutional Review Board of Partners Healthcare System. Asthmatic and non-asthmatic Partners Healthcare patients were identified on the basis of International Classification of Diseases, Ninth Revision (ICD-9) codes for asthma (i.e. those beginning with 493)[22,23]. DNA was obtained from discarded clinical blood samples via the Crimson Project http://www.crimsonproject.org, which identifies discarded Partners Healthcare clinical samples using medical record numbers obtained from the EMR query. For this study, to further ensure that cases truly had asthma, cases (n = 223) were defined as those patients whose EMRs contained an asthma ICD-9 code and whose medication history included usage of at least one beta-agonist or inhaled corticosteroid. Controls (n = 858) were selected as those patients who had been seen in the three years prior to blood collection in at least one of over 850 outpatient clinics but did not have any asthma ICD-9 codes. All patients are white. Using a panel of 187 intergenic SNPs selected randomly throughout the genome[24], and a set of 248 ancestry informative markers[25], we evaluated this cohort for evidence of population stratification. The random panel of 187 SNPs had an association χ^2 ^_187 df _= 168.7, corresponding to a p-value of 0.83. Repeat association testing with adjustment for EIGENSTRAT-derived principal components[26] yielded similar results to the unadjusted results (r^2 ^= 0.95 between corrected and uncorrected association statistics), confirming no significant evidence of population stratification in the iCAP cohort.

### Genotyping and Quality Control

rs5743836 genotyping was performed using a custom design TaqMan 5'→3' assay (ABI, Foster City, California) with the following sequences: Forward primer: GCCTTGGGATGTGCTGTTC; Reverse primer: CAGAGACATAATGGAGGCAAAGGA; VIC Probe: CCTGAAAACTCCC; FAM Probe: CTGGAAACTCCC. The PCR profile consisted of an initial denaturation step at 95°C for 10 min followed by 40 amplification cycles at 95°C for 30 s each. For the PCR reaction, ABI TaqMan Universal PCR Master Mix was used with 6.25 ng of DNA per sample. ABI 7900 Prism was used for imaging.

TLR9 mRNA expression data was extracted from genome-wide gene expression microarray profiles (HumanRef8 v2 BeadChip (Illumina, San Diego, CA)) derived from peripheral blood CD4+ lymphocyte RNA samples in a subset of 200 CAMP participants, as previously reported[27]. TLR9 is assayed on the HumanRef8 v2 array with a 50-mer oligonucleotide with sequence 5'-CAGGGACAACCACCACTTCTATAACCGGAACTTCTGC CAGGGACCCACGG-3', which maps to the terminal coding sequence of the TLR9 reference sequence (NM_017442).

### Statistical Analysis

Family based association testing (FBAT) was performed using PBAT as implemented in HelixTree version 6.4.3 (Golden Helix, Bozeman, Mont)[28]. TLR9-by-vitamin D level interactions were tested using the family-based association tests of interaction (FBATIs) implemented in PBAT[29]. PLINK[30] was utilized for population-based association testing and to derive the transmitted to untransmitted ratios. PLINK was also used for allelic association testing (χ*^2^*) in the case-control study (iCAP). All analyses were performed assuming an additive genetic model.

In CAMP, tests of association with baseline lung function were adjusted for age, sex, and height. Tests of association with all other baseline outcomes were adjusted for age and sex, and all tests for outcomes at the 4 year follow-up time point were also adjusted for treatment group (inhaled steroids vs. placebo or nedocromil). In the population-based analyses in CAMP, we adjusted for population stratification using the top four principal components as derived from genome-wide SNP genotype data[31] using EIGENSTRAT (EIGENSOFT Version 2.0)[26]. SAS version 9.1 (SAS Institute, Cary, NC)was used to manage and analyze the data. We calculated a combined p value (first for all three cohorts and then for the two cohorts of children) using Fisher's exact test[32,33].

### Transient transfection and luciferase assay

Two TLR9 promoter sequences (from -632 to +619) including the SNP of interest (-1237C/T or rs5743836) were amplified by PCR from human genomic DNA and cloned into a pGL3 basic vector at a KpnI and HindIII site. The primers used for PCR were: 5' - GGG GTA CCC CGA GGG GTC ATA TGA GAC TTG GGG GAG TTT TCA - 3' for the 1.2KT and 5'-GGG GTA CCC CGA GGG GTC ATA TGA GAC TTG GGG GAG TTT CCA-3' for the1.2KC promoter. The same reverse primer for both promoters was used: 5' - CCC AAG CTT GCT GGG CTT CTC CAG AGG GT - 3'. For the luciferase reporter assay, 293 cells were seeded into a 12-well plate one day prior to transfection. 300 ng promoter or pGL3 basic vector and 10 ng beta-gal plasmid were transiently transfected into 293 cells using Fugene 6 (Roche Diagnostics, Indianapolis, IN) according to the manufacturer's protocol. 12 hours post transfection, 1,25-dihydroxyitamin D_3 _(Enzo Life Sciences, Farmingdale, NY) was added into cells at various concentrations for an additional 48 hours. Cells were then lysed in 100 ul reporter lysis buffer (Promega, Madison, WI) and the luciferase activity in 50 ul of aliquots of cell lysates was measured using Luciferase Assay System (Promega, Madison, WI) according to the manufacturer's protocol. To monitor the transfection efficiency, a beta-galactosidase assay was conducted using the same lysate according to the published protocol[34]. All constructs were sequenced before transfection and each experiment was independently repeated four times with triplicate wells for each transfection reaction.

## Results

### TLR9 promoter polymorphism genotyping

The TLR9 promoter region SNP rs5743836 was genotyped in 910 families (n = 2261 subjects) in CAMP, in 616 families (n = 1737 subjects) in Costa Rica, and in 1081 subjects from iCAP (223 cases, 858 controls). In CAMP, the genotype quality was high, with a 99.4% completion rate, only one parent-child genotype inconsistency, and perfect concordance upon replicate genotyping of a random 5% subset. In Costa Rica and iCAP, genotype quality was also high with a 98% overall pass rate, perfect concordance upon replicate genotyping of a random 6% subset and only 1 discordant result.

Given the relatively small sample sizes of the nonwhite ethnic groups in CAMP and to avoid false association caused by population stratification, association analyses were restricted to 711 Caucasian probands and their families. Phenotypic characteristics of the Caucasian children from CAMP as well as baseline characteristics of the Costa Rica cohort and iCAP cohorts are shown in Table 1. The minor allele frequency (MAF) for the C allele was 18% in CAMP, 10% in Costa Rica and 15% in iCAP. In all three cohorts parental or control genotypes were in Hardy-Weinberg equilibrium at this locus (CAMP p = 0.29, CR p = 0.78, iCAP p = 0.27).

**Table 1:** Baseline characteristics in each cohort

	CAMP	Costa Rica	iCAP	
		Mean (SD)		
			Cases	Controls
N	711	616	223	858
Age	8.8 (2.1)	9.0 (1.8)	25.5 (7.0)	26.1 (5.3)
Sex (N, % male)	416 (59%)	370 (60%)	43 (19%)	136 (16%)
Baseline post-bronchodilator FEV_1_	1.82 (0.5)	1.83 (0.5)		
baseline post-bronchodilator FEV_1 _percent-predicted	104 (12.6)	104 (16.1)		
Baseline post-bronchodilator FVC	2.15 (0.63)	2.14 (0.57)		
Baseline post-bronchodilator FVC percent-predicted	107 (12.6)	109 (15.8)		
Post-bronchodilator FEV_1 _at 4 yr follow up	2.79 (0.79)			
Post-bronchodilator FEV_1 _at 4 yr follow up percent predicted	103 (12.6)			
Post-bronchodilator FVC at 4 yr follow up	3.35 (0.98)			
Post-bronchodilator FVC at 4 yr follow up percent predicted	107 (12.2)			
				
rs5743836 C: minor allele T: major allele				
**Minor allele frequency**	18%	10%	15%	

### Association testing of TLR9 promoter region polymorphism and asthma

Though there was a trend towards over-transmission of the minor allele (C) from parent to asthmatic offspring in the CAMP cohort, (transmitted to untransmitted ratio [T:U] of 136:112, ratio of 1.21 for the C allele), the association was not statistically significant (FBAT p = 0.14). There was no trend towards an association in either Costa Rica (FBAT p = 0.77) or iCAP (χ^2 ^p = 0.83); see Table 2. Using Fisher's exact test, we calculated a combined p value first for all three cohorts together, and separately for the two childhood cohorts; neither of these values was statistically significant (p = 0.57 for all three, p = 0.35 for the two childhood cohorts).

**Table 2:** Results for association testing with rs5743836 and asthma affection status

Cohort	**Sample size**^**1**^	**p value**^**2**^
**CAMP**	208	0.14
**Costa Rica**	185	0.77
**iCAP cohort**	cases: 223 ctrls: 858	0.83

^1^Number of informative families in CAMP and Costa Rica; N for iCAP

^2^FBAT in CAMP and Costa Rica, chi-square test in iCAP

Moreover, with the exception of post-bronchodilator measures of lung function (see below), no associations were noted with intermediate phenotypes analyzed in CAMP - including bronchodilator response to albuterol, airways responsiveness, total IgE, skin prick tests for common aeroallergens, and blood eosinophil counts - suggesting that TLR9 genotype is not a major determinant of asthma in the CAMP cohort.

**Figure 1:**
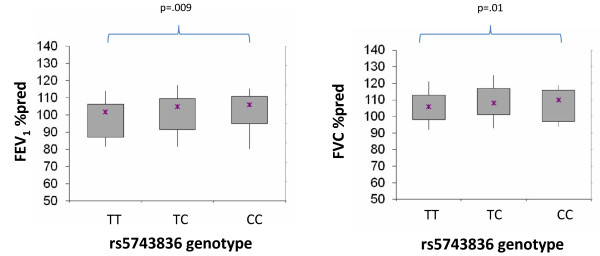
**Results from population-based analysis of rs5743836 genotype and lung function measures in CAMP**. Median values (X) and interquartile range for FEV_1 _and FVC at the 4 year follow up timepoint by rs5743836 genotype in CAMP.

Association of TLR9 was noted with post-bronchodilator lung function measures (FEV_1 _and FVC) at the 4-year follow-up time point in CAMP, with each additional copy of the minor allele associated with higher lung function (p = 0.003 and p = 0.008, respectively). The observed associations were robust to multivariate adjustment for age, height, sex and treatment arm. Similar nominal associations were noted when modeled using population-based approaches (p = 0.009 and p = 0.01, respectively; Figure 1). However, neither the family- nor population-based tests were significant after adjustment for multiple comparisons, and the small actual difference in values was of uncertain clinical significance. Associations were noted only with year 4 measurements, but not with baseline measures, and were not replicated in the Costa Rica cohort (data not shown) raising questions about the importance of the observed associations. Moreover, the TLR9 promoter variant was not associated with asthma exacerbations over the 4 years of the clinical trial (defined as emergency room visits, hospitalizations or prednisone bursts).

### Interaction with Vitamin D

Based on prior knowledge of the interaction of TLR9 with vitamin D[12], and the relationship to induction of IL-10 producing Tregs, we tested for an interaction between the SNP and vitamin D levels in this cohort. We did not find a significant association with this SNP and vitamin D insufficiency (defined as ≤30 ng/ml) with any lung function measures (p = 0.44-0.98 for interaction), nor with the other outcomes tested including those listed above. We looked at vitamin D levels as a continuous variable as well and found similar results (data not shown).

### Functional studies of TLR9 promoter region genotype

**Figure 2:**
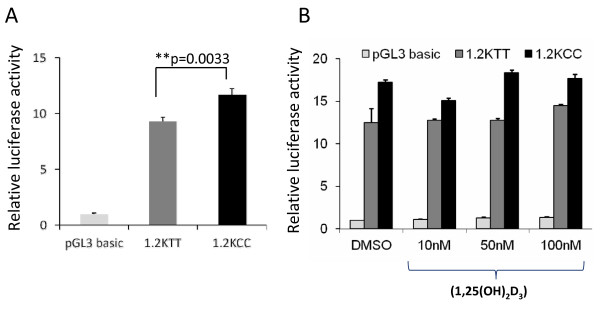
**TLR9 promoter activity in HEK 293 cells**. A. Higher TLR9 promoter activity was associated with CC allelic variant at rs5743836. HEK 293 cells were transiently transfected with reporter constructs with TLR9 promoter containing either genotype TT (1.2KTT) or CC (1.2KCC) at rs5743836. 48 hours post transfection, cells were lysed for luciferase activity and normalized to β-gal readings. Luciferase activity from pGL3-basic vector transfected cells was defined as 1. Standard deviation is from triplicate wells. Similar results were obtained from at least three independent experiments. B. Vitamin D has no significant effect on the promoter activity of TLR9. HEK 293 cells were transfected with 1.2KTT and 1.2KCC TLR9 promoter. 12 hours post transfection, cells were treated with active form of Vitamin D (1,25(OH)_2_D_3_) at various concentrations. 48 hours later, promoter activity was analyzed by luciferase assay. Standard deviation is from triplicate wells of each transfection.

To test whether rs5743836 genotype affects promoter activity of the TLR9 gene, we cloned two promoters containing either the TT or CC genotype into firefly luciferase reporter vectors; the two promoters were designated as1.2KT and 1.2KC respectively. Promoter activity was assessed using the luciferase assay system. After standardization with beta-gal, the promoter containing the T variant (1.2KT) showed lower activity than the promoter containing the C variant (9.3 fold versus 11.7 fold, P = 0.003)

(Figure 2A). We further investigated whether the active form of vitamin D (1,25(OH)_2_D_3_) affects promoter activity of TLR9. After 2 days of treatment with 1,25(OH)_2_D_3_, the change of TLR9 promoter activity was negligible in 293 cells (Figure 2B).

Because the CC genotype was associated with higher promoter activity, we analyzed TLR9 mRNA expression levels from CD4+ lymphocytes from a subset of 200 non-Hispanic white CAMP asthmatics. Expression levels were not significantly associated with rs5743836 genotype (FBAT p = 0.80, population-based p = 0.38).

## Discussion

Based on previous studies by our group[5], and knowledge of a potential interaction between TLR9 and vitamin D[12,35], we examined possible associations between rs5743836, a SNP in the promoter region of TLR9, and asthma in three different cohorts of asthmatics, as well as with related quantitative phenotypes in the CAMP cohort. We also looked for an interaction between this SNP and vitamin D levels, and performed functional studies using this SNP. Although there was a trend towards over transmission of the C allele in children with asthma in the CAMP cohort, this was not statistically significant; nor was it found in the other two cohorts of asthmatics. We found a weak association between this SNP and lung function measures in CAMP at the 4-year follow-up point in both the family- and population-based analyses. However this was no longer statistically significant once corrected for multiple comparisons, was only observed at one of two time points studied, and was not replicated in the Costa Rican cohort. We found no other significant associations between this SNP and any other intermediate phenotypes tested, nor was there an interaction with vitamin D levels. Though we demonstrated *in vitro *evidence that the rs5743836 CC genotype confers increased expression of TLR9, this association was not observed in an epidemiological context: the variant was not associated with CD4+ lymphocyte TLR9 expression in the CAMP cohort. Together, these data suggest this polymorphism does not play a role in susceptibility to asthma in these three cohorts, nor in the development of asthma-related phenotypes in CAMP.

Evidence from prior studies suggests that this SNP in TLR9 could be important in asthma and other inflammatory diseases. Our group had previously found the C allele of this SNP to be associated with asthma in European Americans[5]. Another study showed similar findings in a cohort of Tunisian children with asthma, with higher risk conferred by the C allele[6]. The SNP has been found to be associated with other allergic diseases such as ABPA in a population from the UK[7]; one of the diagnostic criteria for ABPA is the presence of asthma. An association was also found in a study of atopic eczema in Germans[8], though it was the T allele that was over transmitted in affected individuals. In addition, it has been found in association with IBD[9-11,36] as well as with Hodgkin's disease[37] susceptibility to tuberculosis infection[38] and viral set point in HIV[39].

Furthermore, other emerging data about TLR9 more generally offers compelling evidence for its role in modulation of inflammatory and allergic diseases, and a possible route to therapy. TLR9 knockout mice have lower airways hyperresponsiveness in a model of fungal asthma[40]. Additionally, the ligand for TLR9 (CpG) is being investigated in animal models and some human studies as an adjuvant for immunotherapy of allergic diseases including allergic rhinitis and asthma[3,41-43]. Although the exact mechanism of benefit is unknown, the inhibition of TH2 cytokines may be achieved through induction of TH1-type cytokines such as IL12, IFNα, IFNγ, or through other mechanisms[44,45] including induction of IL-10 or indoleamine 2,3-dioxygenase activity and Treg cells[46,47]. The biologic effects of TLR9 activation on allergic inflammation are likely highly complex and merit further investigation.

However, conflicting studies showed no association with this SNP or others in TLR9 in similar diseases. No association was found with atopy in a second German cohort[48]. The rs5743836 polymorphism was not observed in a sequencing survey of 32 Japanese asthmatics. Other TLR9 variants that were observed in that cohort showed no associations with asthma affection status or serum IgE level[49]. Similar studies in Caucasian populations from Canada and Australia[50], and in French Europeans[51] also failed to observe associations with asthma. TLR9 variation does not appear to have association with either SLE[52-54] or Behcet's disease[55,56].

The strengths of our study include the use of three different asthmatic populations: two in children and one in adults. In addition, our analysis of intermediate phenotypes was carried out in CAMP, a well-characterized population of asthmatics with extensive phenotyping data including lung function measures (spirometry, methacholine challenge results, bronchodilator responsiveness), skin test results, IgE and eosinophil levels, and data on exacerbation frequency. We used both a family-based association test, which is robust to population stratification, as well as population-based testing, which is more powerful than family-based testing. Population based tests were adjusted for population stratification using principal components[26]. The addition of a high quality assessment of functionality with this SNP strengthens any possibility of association.

There are several possible reasons for discrepant results among studies of association between this SNP and asthma. These include sample size, differing phenotype definitions, differing populations, population stratification, differing linkage disequilibrium patterns, and genetic and environmental heterogeneity. Our study includes two well-characterized cohorts of children with asthma, and a cohort of adult asthmatics. Asthmatics of differing severity or age may be a different enough phenotype to lead to different results from association testing. We may have been limited in power to detect an association given the sample sizes in the three cohorts. In addition, the population-based association testing performed in CAMP examined variability *among *individuals within the cohort of asthmatics; if we had compared our cohort to a well-matched control group, testing intermediate phenotypes may have revealed an association. Prior studies of this SNP and asthma were small and were not controlled for population stratification. In the Tunisian study, significant results from the case-control analysis were not reproduced when families were analyzed using the transmission disequilibrium test (TDT) which is robust to population stratification, suggesting positive results from the case-control portion may have been spurious.

We detected a moderate increase in promoter activity associated with the CC genotype. This could be explained by the possible creation of an NF-κB binding site due to a change to the C allele which has been shown in *in silico *analysis[57]. Higher promoter activity with the T allele has been found in one previous study[8] though more recently another study showed higher activity with the C allele, consistent with our findings[58]. Since we found no correlation between rs5743836 genotype and TLR9 mRNA level in a subset of the CAMP population, we cannot exclude the existence of other functional variants in this region that interact with rs5743836 to regulate the expression of TLR9. Given the complexity of gene regulation *in vivo*, the lack of association with TLR9 expression levels is not surprising.

## Conclusions

In summary, we examined associations between a SNP in the promoter of TLR9, rs5743836, and asthma in three unrelated cohorts of asthmatics, and found no statistically significant associations after correcting for multiple testing. In addition, we performed functional studies which showed that this SNP does appear to have functional significance. Despite these *in vitro *findings, our results suggest that this polymorphism does not play a significant role in asthma susceptibility or asthma related phenotypes. This does not rule out a role for the gene in asthma. Despite these negative results, future studies to further elucidate the effects of TLR9 activation in modulating inflammation, as well as the possible role of TLR9 agonists in therapy for allergic and inflammatory diseases are warranted.

## Competing interests

Dr. Soto-Quiros reports receiving lecture fees from AstraZeneca, GlaxoSmithKline, and Merck Sharp & Dohme; Dr. Avila reports receiving lecture fees from AstraZeneca and Merck Sharp & Dohme; Dr. Hawrylowicz reports receiving lecture honoraria/fees from Merck. Dr. Raby reports receiving lecture fees from Novartis Pharmaceuticals for in-house educational services. No other authors have any competing interests to declare.

## Authors' contributions

NEL contributed to the study design, performed the analyses and drafted the manuscript. XZ performed the functional studies and contributed to the manuscript writing. JL-S contributed to the study design and statistical analysis. BEH contributed to the statistical analysis. RL contributed to the statistical analysis. MSQ contributed to the study design and data collection. LA contributed to the study design and data collection. JCC contributed to the study design and provided critical revision of the manuscript. CMH contributed to the data analysis and interpretation. BAR contributed to the study design and provided critical revision of the manuscript. AAL contributed to the study design and provided critical revision of the manuscript. All authors read and approved the final manuscript.

## Funding

CAMP: The CAMP Genetics Ancillary Study is supported by U01 HL075419, U01 HL65899, P01 HL083069 and T32 HL07427 from the National Heart, Lung and Blood Institute, National Institutes of Health (NIH/NHLBI). The expression work is supported by grant R01 HL086601 from the (NIH/NHLBI). Dr. Litonjua is supported by R01 AI056230. Dr. Lazarus is supported by R01 HG0036.

Costa Rica: The Genetics of Asthma in Costa Rica study is supported by NIH grants HL04370 and HL66289.

iCAP: This work was supported by the NIH grant 5U54LM008748-02 (National Centers for Biomedical Computing).

## Pre-publication history

The pre-publication history for this paper can be accessed here:

http://www.biomedcentral.com/1471-2350/12/26/prepub
